# Changes in relative peripheral refraction and optical quality in Chinese myopic patients after small incision lenticule extraction surgery

**DOI:** 10.1371/journal.pone.0291681

**Published:** 2023-10-04

**Authors:** Yuqin Du, Yuehua Zhou, Mingwei Ding, Mingxu Zhang, Yujuan Guo

**Affiliations:** 1 Eye School of Chengdu University of TCM, In eye Hospital of Chengdu University of TCM, Chengdu, China; 2 Beijing Ming Vision and Ophthalmology, Dongcheng District, Beijing, China; UNSW: University of New South Wales, AUSTRALIA

## Abstract

**Purpose:**

To observe changes in retinal refraction difference values (RDV) and aberrations after small incision lenticule extraction (SMILE) surgery and evaluate their correlations.

**Methods:**

This study recruited 112 patients (112 eyes) who underwent SMILE for myopia. Participants were classified into the Low and Moderate Myopia group (LM, -0.50 to -6.0 D) and High Myopia group (HM, >-6.0 D) according to the central spherical equivalent (SE). RDVs in the five retinal eccentricities from 0° to 10°, 10° to 20°, 20° to 30°, 30° to 40°, and 40° to 53° are recorded as RDV-(0–10), RDV-(10–20), RDV-(20–30), RDV-(30–40), and RDV-(40–53), respectively; additionally, RDVs have four sectors, i.e., RDV-Superior (RDV-S), RDV-Inferior (RDV-I), RDV-Temporal (RDV-T), and RDV-Nasal (RDV-N). With a 3-month follow-up, changes in RDV (ΔRDV) and changes in aberrations [Δtrefoil, Δcoma, Δspherical aberration (SA), and Δtotal higher-order aberrations (HOA)] after surgery were recorded.

**Results:**

No significant differences were observed in total RDV (TRDV), RDV-(0–53), RDV-S, RDV-I, RDV-N, trefoil, coma, and SA between the two groups before SMILE surgery. However, after SMILE, hyperopic defocus values [TRDV, RDV-(20–53), RDV-S, RDV-T, and RDV-N] in the LM group and hyperopic defocus values [TRDV, RDV-(20–53), RDV-S, and RDV-N] in the HM group were significantly lower at 3 months postoperatively than preoperatively, and the RDV-(40–53), RDV-S, and RDV-N were lower in the HM group than in the LM group. Aberrations [trefoil (vertical), coma, and HOA] in the LM group and aberrations (trefoil, coma, SA, and HOA) in the HM group were significantly higher at 3 months postoperatively than preoperatively, and the coma, trefoil(horizontal), SA, and HOA were higher in the HM group than in the LM group. In the multivariate analysis, ΔRDV-(40–53) was significantly correlated with ΔSA, and ΔRDV-T and ΔRDV-N were significantly correlated with Δcoma (horizontal).

**Conclusions:**

Our findings suggest that SMILE reduces retinal peripheral hyperopic defocus but introduces some higher-order aberrations, especially in people with high myopia refractive errors.

## Introduction

Myopia has become a global health problem, and its prevalence continues to increase every year [1]. In recent years, many clinical studies have found that visual signals from the peripheral retina may induce myopia [2], which is an important topic in the research field of vision. In addition, some animal experiments have confirmed that hyperopic defocus stimulates axial length (AL) growth, while myopic defocus inhibits AL growth [3–6]. The effect of optical defocus on the control of the AL depends on the degree of retinal peripheral defocus [7, 8]. Therefore, peripheral refraction status, especially relative hyperopic defocus, has an important effect on the growth of the AL and the progression of refractive error. Notably, multispectral refractive topography (MRT), an ophthalmic device that can detect retinal refraction status rapidly with good repeatability and accuracy, can help to accurately assess the status of retinal peripheral defocus [9, 10]. Small incision lenticule extraction (SMILE) is a newer refractive correction procedure that maintains the connection between the corneal epithelium and the Bowman’s layer, and clinical studies have shown favorable results in terms of safety, efficacy, stability, and predictability in the correction of myopia [11–14].

However, the changes of peripheral refraction in different eccentricity ranges of the retina caused by SMILE remain unclear. Accordingly, in this study, we measured the refraction difference value (RDV) in different parts of the retina before and after SMILE surgery in Chinese myopic patients using MRT and investigated the relationship between changes in RDV and those in aberrations. The present findings would provide some reference and theoretical basis for the personalized design of corneal refractive surgery and the prevention and control of myopia in adolescents.

## Materials and methods

### General information

One hundred and twelve adults who underwent SMILE for myopia in Beijing Ming Vision and Ophthalmology (a clinic practice hospital of In eye Hospital of Chengdu University of TCM) from October 9, 2022 to January 10, 2023. This study was conducted in accordance with the principles of the Declaration of Helsinki, and written informed consent was obtained from all participants. Ethical approval was obtained from the Institutional Review Board of the In eye Hospital of Chengdu University of TCM (approval number: 2022yh-022).

### Inclusion and exclusion criteria

Inclusion criteria were as follows: (1) age of 18–48 years; (2) annual refractive increase of ≤0.50 D, stable for ≥2 years; (3) corrected distance visual acuity (CDVA) logMAR ≤ 0.10; (4) spherical soft contact lens discontinued for more than 14 days, toric soft contact lens and rigid gas permeable contact lens discontinued for more than 1 month, and orthokeratology (OK) lenses discontinued for more than 3 months; (5) no relevant contraindications to surgery; (6) the patient and family members all provided informed consent to the treatment plan and voluntarily signed an informed consent form. Exclusion criteria were as follows: (1) diagnosed or suspected keratoconus; (2) patients with insufficient follow-up data within 3 months after surgery.

Participants were classified into two refractive groups according to central spherical equivalent (SE) refractive error: Low and Moderate Myopia group (LM, -0.50 to -6.0 D) and High Myopia group (HM, >-6.0 D).

### Preoperative routine examination

**Fig 1:**
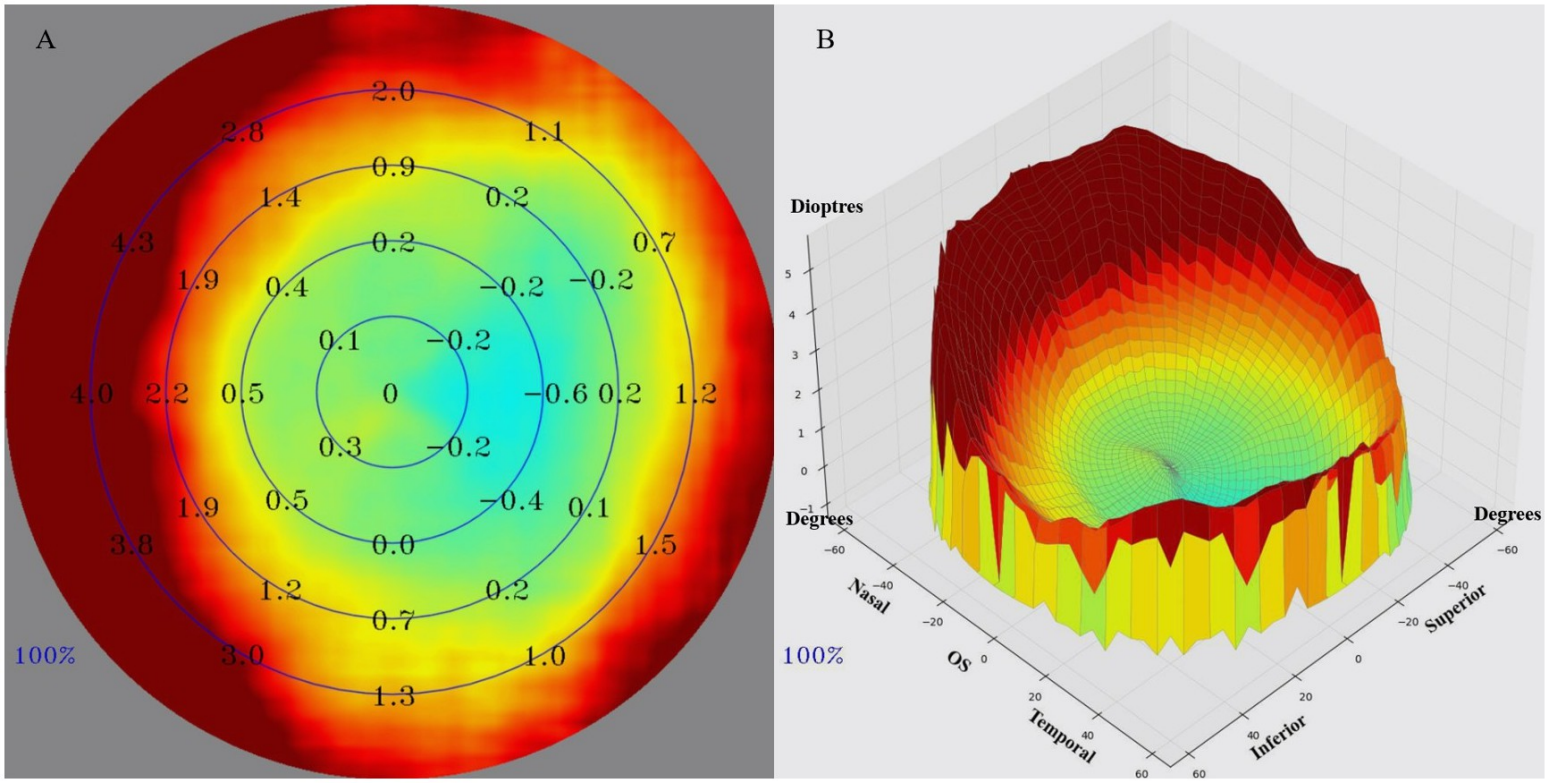
(A) Retinal refraction difference value (RDV) findings of multispectral refractive topography are presented: the innermost circle, RDV-(0–10); the first ring area, RDV-(10–20); the second ring, RDV-(20–30); the third ring area, RDV-(30–40); and the fourth ring area, RDV-(40–53). (B) A three-dimensional image of the relative refractive state of the central and peripheral retina.

All patients underwent preoperative routine ophthalmic examinations at the Refractive Surgery Center to determine whether they had good ocular health and were suitable for the surgical requirements. AL and central corneal thickness were measured using an optical biometer (LS900, Haag-Streit AG, Switzerland). Topography of both eyes was measured using a corneal topographer (TMS-4, Tomey, Japan), and corneal curvature steep keratometry, flap keratometry, and average keratometry were recorded. MRT (MSIC2000, ShengDa TongZe, Shenzhen, China) was used to measure the retinal RDV (Fig 1) and record the retinal total refraction difference value (TRDV) from the fovea to 53 degrees. RDVs in the five retinal eccentricities from 0° to 10°, 10° to 20°, 20° to 30°, 30° to 40°, and 40° to 53° were recorded as RDV-(0–10), RDV-(10–20), RDV-(20–30), RDV-(30–40), and RDV-(40–53), respectively. RDVs also had four sectors, including RDV-Superior (RDV-S), RDV-Inferior (RDV-I), RDV-Temporal (RDV-T), and RDV-Nasal (RDV-N). The hyperopic RDV is represented in the results by positive values, while the myopic RDV is represented by negative values. Wavefront aberration measurements were performed with an iTrace analyser (TX77060, Houston, Inc., USA) to obtain the vertical trefoil, vertical coma, horizontal coma, horizontal trefoil, and spherical aberration (SA), and the root mean square of total higher-order aberrations (HOA) was calculated using Zernike polynomials at a 5.0 mm analysis diameter. In addition, 0.5% compound tropicamide was used to induce cycloplegia, and computerized optometry, retinoscopy optometry, and subjective optometry were then used to measure the refractive power of the subjects and calculate the equivalent spherical equivalent (SE): SE = Dioptre Sphere +1/2 Dioptre Cylinder.

### Surgical procedure

All patients were operated on by the same surgeon (YueHua Zhou) with extensive experience in SMILE. The Visumax Femtosecond Laser System (Carl Zeiss Meditec AG, Jena, Germany) was set to the expert mode with a repetition rate of 500 kHz. Additional parameter settings were as follows: pulse energy, 130 nJ; cap thickness, 100–110 μm; optical zone diameter, 6.5 mm; cap diameter, 7.5 mm; and base thickness, 10 μm. The upper side incision was set at 12:00 o’clock, and the width was 2.0 mm. Patients were instructed to gaze at the fixation lamp, and negative pressure was applied to flatten and fix the eye. Laser scanning and blasting were used to make a lenticule, and the lenticule was then separated and removed.

### Postoperative follow-up

The postoperative follow-up was 3 months, and the following parameters were assessed: uncorrected distance visual acuity (UDVA), CDVA, dioptre, RDV, corneal topography, aberrations, slit lamp, and fundus. Changes in RDV (ΔRDV) and aberrations (Δtrefoil, Δcoma, ΔSA, and ΔHOA) were also recorded: ΔRDV = postoperative RDV minus preoperative RDV; Changes in aberrations (Δtrefoil, Δcoma, ΔSA, and ΔHOA) = postoperative aberrations minus preoperative aberrations.

### Statistical analysis

R language (version 4.3.0) was used for statistical analysis. Kolmogorov–Smirnov normality test was used to determine whether the data conformed to a normal distribution. In the baseline analysis and analysis of the differences between the LM and HM groups, Levene’s test was used to test the homogeneity of variance for data that passed the normality test. The independent sample t-test was used for data with homogeneous variance, and the welch-t test was used for data with non-homogeneous variance. The Mann–Whitney U test was used for data that did not pass the normality test. Gender differences were tested by chi-square test. For the analysis of intra-group differences between the preoperative and postoperative changes in each group, the paired t-test, paired welch-t test, and paired Mann–Whitney U test were used according to their data distribution and variance homogeneity. To further analyze the relationship between preoperative and postoperative changes in retinal defocusing status and changes in aberrations, correlation analysis and linear regression analysis were performed. Pearson correlation coefficients were used for normally distributed data, and Spearman correlation coefficients were used for non-normally distributed data; further, simple linear regression and multiple regression analyses were used to establish possible relationship equations between them. Normally distributed data are expressed as mean ± standard deviation, and non-normally distributed data are presented using median (Q1, Q3). A p-value <0.05 indicated that the difference was statistically significant.

## Results

### Descriptive statistics of the participants

In total, 112 participants (38 men and 74 women; aged 18–42 years; 112 eyes, only right eyes were considered) were included in the study. The LM group had 66 participants (66 eyes), and the HM group had 46 participants (46 eyes). No statistically significant differences were observed between the two groups in terms of gender, age, central corneal thickness, average keratometry, and CDVA (all p >0.05) (Table 1). The median SE and AL were -4.51±0.99 and 25.49±0.90 mm, respectively, in the LM group and -7.04±0.60 and 26.13±0.91 mm, respectively, in the HM group. The differences in SE and AL between the two groups were statistically significant (all p <0.05) (Table 1).

**Table 1:** Baseline comparison between the LM and HM groups.

Parameters	LM group (n = 66)	HM group (n = 46)	χ^2^/t	p
Gender (*n/n*)				
Female	43(38.39%)	31(27.68%)	0.0019	0.9653
Male	23(20.54%)	15(13.39%)		
Age(years)	28.65±6.66	27.63±5.79	0.8412	0.4021
Equivalent spherical (D)	-4.51±0.99	-7.04±0.60	16.8627	<0.0001*
Central corneal thickness (μm)	530.35±25.60	535.96±18.61	-1.3420	0.1824
Average keratometry (D)	43.70±1.43	43.85±1.62	-0.5378	0.5918
AL (mm)	25.49±0.90	26.13±0.91	-3.6837	0.0004*

Note: D, diopter; AL, axial length; LM, Low and Moderate Myopia; HM, High Myopia;

* P<0.05.

### Changes in RDV after SMILE surgery

**Fig 2:**
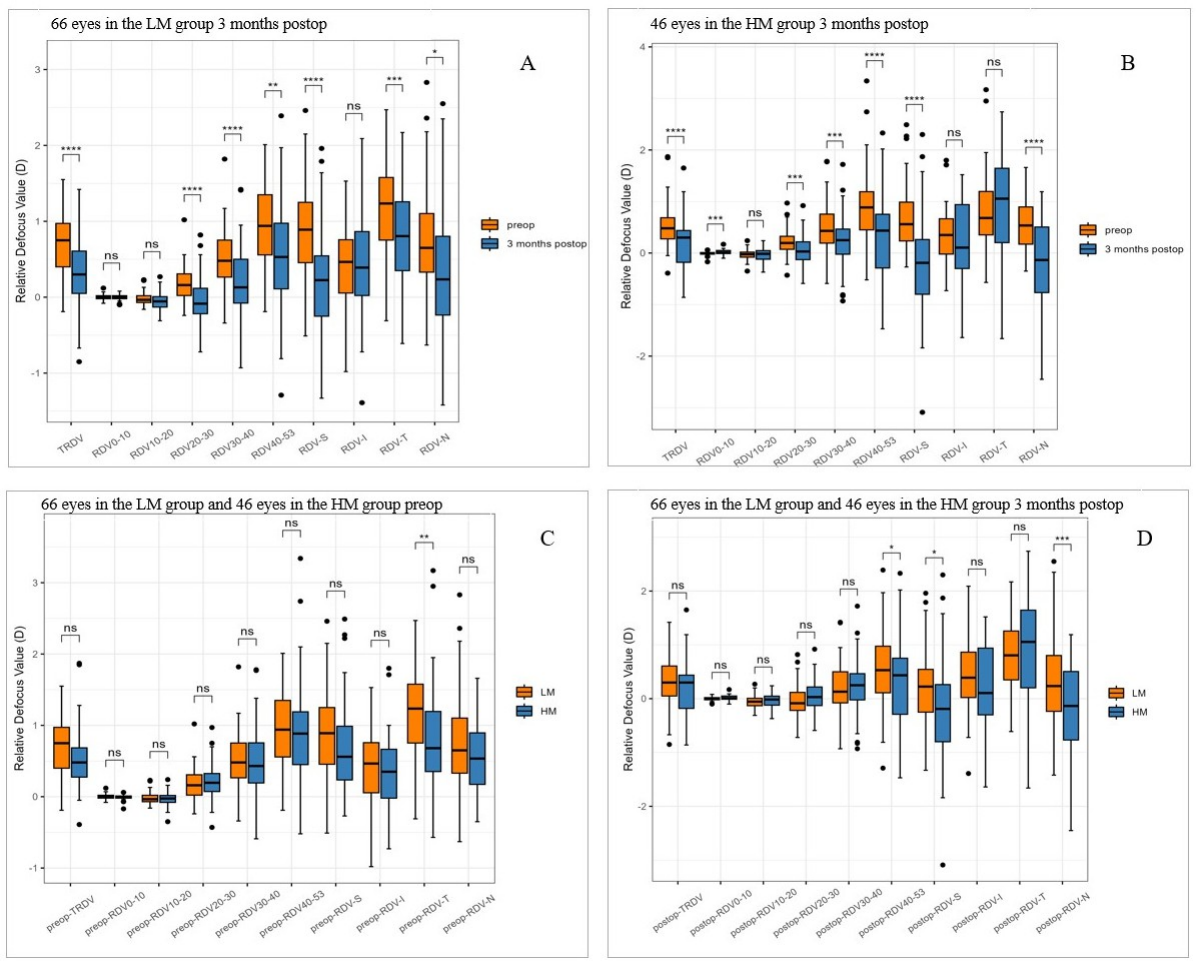
RDV at different eccentricity rates preoperatively versus 3 months postoperatively in the LM group (A) and HM group (B). Preoperative differences (C) and postoperative differences (D) in RDV between the two groups. LM, Low and Moderate Myopia; HM, High Myopia; RDV, refraction difference value; RDV-I, RDV-inferior; RDV-N, RDV-nasal; RDV-S, RDV-superior; RDV-T, RDV-temporal; TRDV, total RDV of 0 to 53°. Ns: P≥0.05, * P<0.05, ** P<0.01, *** P<0.001, **** P<0.0001.

All the mean values of RDV-(20–53), RDV-S, RDV-I, RDV-T, and RDV-N indicated hyperopic defocus in preoperative myopic patients. In the LM group, all hyperopic defocus values, i.e., TRDV, RDV-(20–53), RDV-S, RDV-T, and RDV-N, were significantly lower at 3 months postoperatively than preoperatively (all p <0.05) (Fig 2A). In the HM group, all hyperopic defocus values, i.e., TRDV, RDV-(20–53), RDV-S, and RDV-N, were significantly lower at 3 months postoperatively than preoperatively (all p <0.05) (Fig 2B). However, the RDV-(0–10) was mildly higher at 3 months postoperatively than preoperatively in the HM group (t = -3.8585, p = 0.0004) (Fig 2B). No significant differences in TRDV, RDV-(0–53), RDV-S, RDV-I, and RDV-N before the operation were observed between the LM and HM groups (all p>0.05), and only the RDV-T before the operation was higher in the LM group than in the HM group (Fig 2C). At 3 months postoperatively, the RDV-(40–53), RDV-S, and RDV-N were lower in the HM group than in the LM group (all p <0.05) (Fig 2D).

### Changes in aberrations after SMILE surgery

**Fig 3:**
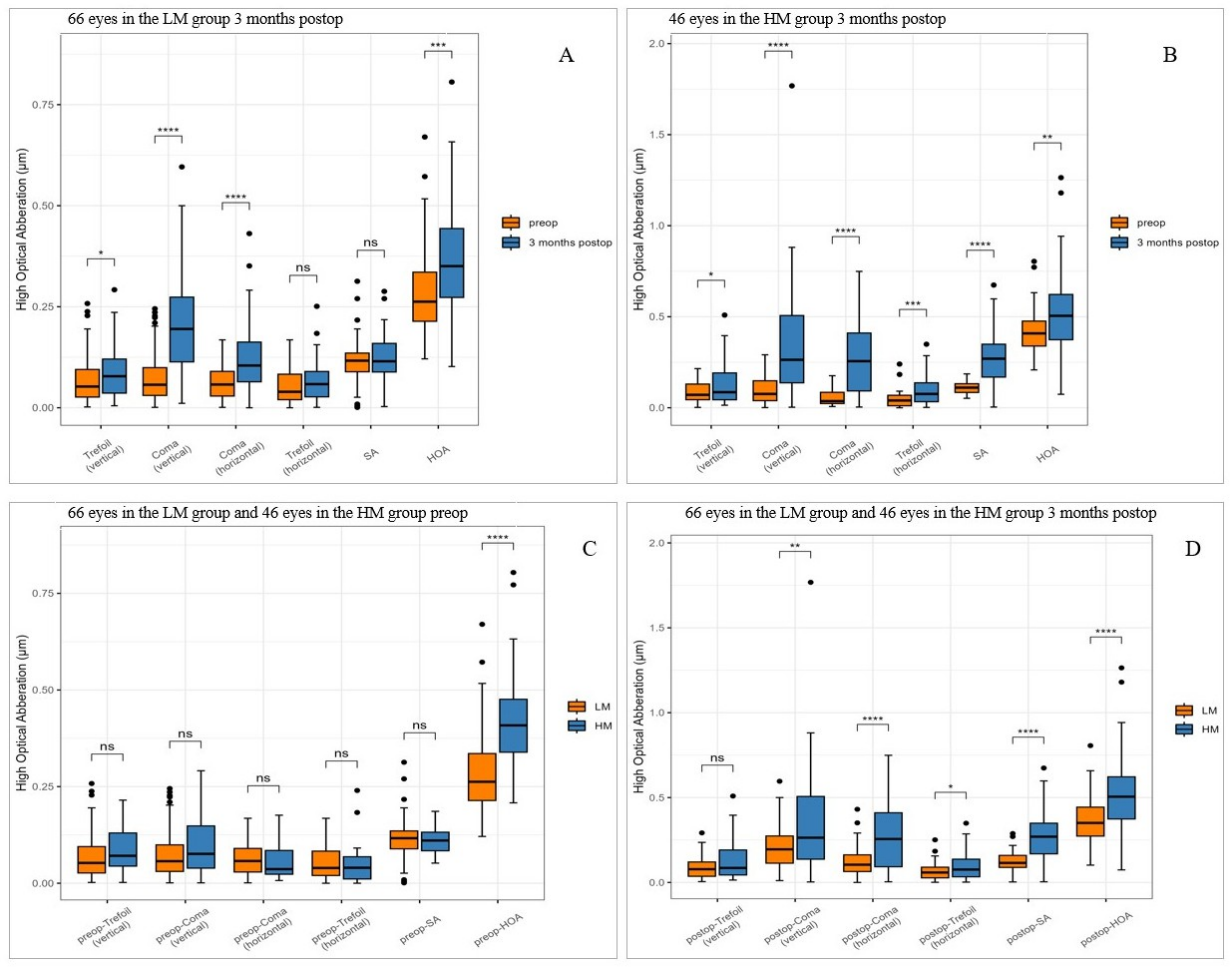
Aberrations for a 5 mm pupil preoperatively versus 3 months postoperatively in the LM group (A) and HM group (B). Preoperative differences (C) and postoperative differences (D) in aberrations between the two groups. LM, Low and Moderate Myopia; HM, High Myopia. SA, spherical aberration; HOA, total higher-order aberrations. Ns: P≥0.05, * P<0.05, ** P<0.01, *** P<0.001, **** P<0.0001.

In the LM group, the trefoil (vertical), coma (vertical), coma (horizontal), and HOA were significantly higher at 3 months postoperatively than preoperatively (all p < 0.05) (Fig 3A). In the HM group, the trefoil, coma, SA, and HOA were significantly higher at 3 months postoperatively than preoperatively (all p < 0.05) (Fig 3B). No significant differences in trefoil, coma, and SA before the operation were observed between the LM and HM groups (all p>0.05), and the HOA before the operation was higher in the HM group than in the LM group (t = -5.4864, p <0.0001) (Fig 3C). At 3 months postoperatively, coma, trefoil (horizontal), SA, and HOA were higher in the HM group than in the LM group (all p <0.05) (Fig 3D).

### Relationship between changes in RDV (ΔRDV) and changes in aberrations

**Fig 4:**
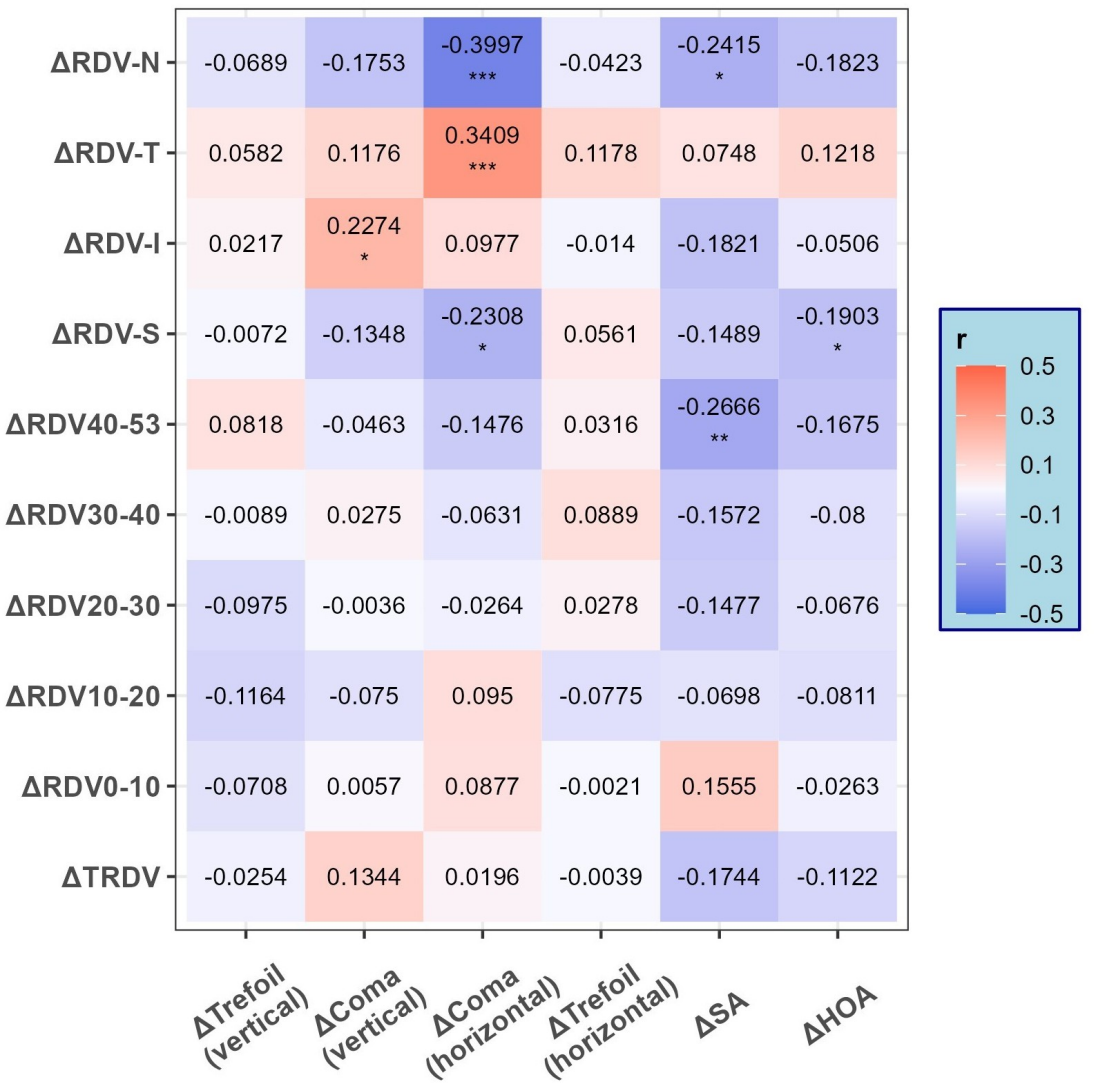
Correlation of ΔRDV with changes in aberrations. Δ, postoperative value minus preoperative value; RDV, refraction difference value; RDV-I, RDV -inferior; RDV-N, RDV-nasal; RDV-S, RDV-superior; RDV-T, RDV-temporal; TRDV, total RDV of 0 to 53°; SA, spherical aberration; HOA, total higher-order aberrations. * P<0.05, ** P<0.01, *** P<0.001.

The right eyes of all participants were included in this study. In the correlation analysis between ΔRDV and changes in aberrations (Δtrefoil, Δcoma, ΔSA, and ΔHOA) at 3 months postoperatively (Fig 4), Δcoma (vertical) showed a positive correlation with ΔRDV-I (r = 0.2274, p = 0.0159); Δcoma (horizontal) showed a positive correlation with ΔRDV-T (r = 0.3409, p = 0.0002) and a negative correlation with ΔRDV-N (r = -0.3997, p <0.0001) and ΔRDV-S (r = -0.2308, p = 0.0144); ΔSA was negatively correlated with ΔRDV-(40–53) (r = -0.2666, p = 0.0045) and ΔRDV-N (r = -0.2415, p = 0.0103); and ΔHOA was negatively correlated with ΔRDV-S (r = -0.1903, p = 0.0445) (Fig 4).

**Fig 5:**
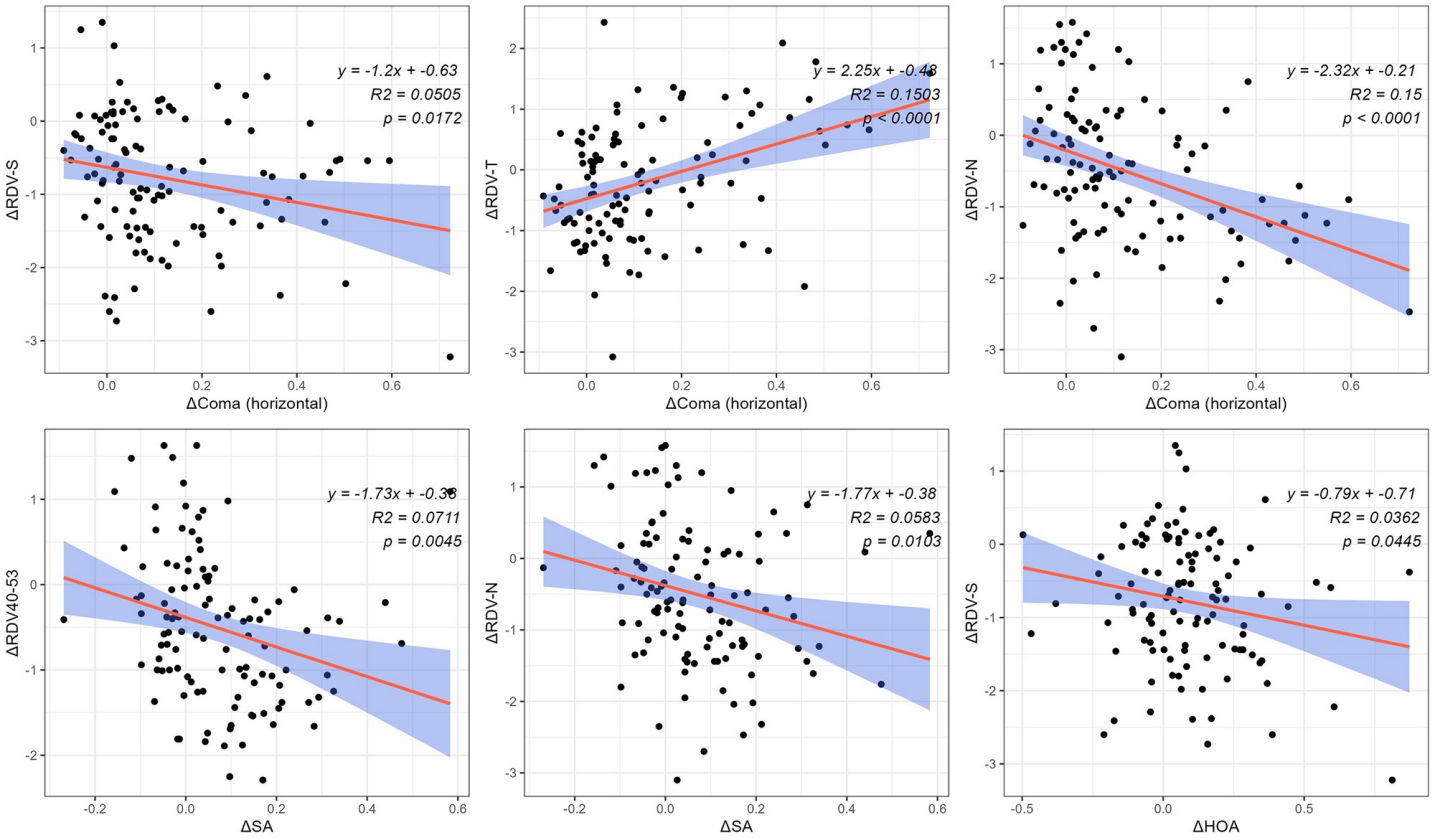
Simple linear regression of ΔRDV with changes in aberrations. Δ, postoperative value minus preoperative value; RDV, refraction difference value; RDV-I, RDV -inferior; RDV-N, RDV-nasal; RDV-S, RDV-superior; RDV-T, RDV-temporal; TRDV, total RDV of 0 to 53°; SA, spherical aberration; HOA, total higher-order aberrations.

In the simple linear regression, ΔRDV-S decreased significantly with Δcoma (horizontal) (r2 = 0.0505, slope = -1.20, p = 0.0172) and ΔHOA (r2 = 0.0362, slope = -0.79, p = 0.0445); ΔRDV-T increased significantly with Δcoma (horizontal) (r2 = 0.1503, slope = 2.25, p <0.0001); ΔRDV-N decreased significantly with Δcoma (horizontal) (r2 = 0.150, slope = -2.32, p <0.0001) and ΔSA (r2 = 0.0583, slope = -1.77, p = 0.0103); and ΔRDV-(40–53) decreased significantly with ΔSA (r2 = 0.0711, slope = -1.73, p = 0.0045) (Fig 5).

In the multivariate analysis of ΔRDV (ΔTRDV, ΔRDV-(0–53), ΔRDV-S, ΔRDV-I, ΔRDV-T, ΔRDV-N) using multiple linear regression, changes in aberrations (Δtrefoil, Δcoma, ΔSA, and ΔHOA) were included as independent variables (Table 2). ΔRDV-(40–53) was significantly correlated with ΔSA (standardized coefficient: *Beta* = -0.2909, *p* = 0.0061), ΔRDV-T was significantly correlated with Δcoma (horizontal) (standardized coefficient: *Beta* = 0.4028, *p* = 0.0001), and ΔRDV-N was significantly correlated with Δcoma (horizontal) (standardized coefficient: *Beta* = -0.3421, *p* = 0.0007) (Table 2).

**Table 2:** Multivariate analysis of the association between changes in RDV (ΔRDV) and changes in aberrations at 3 months postoperatively.

	Unstandardized Coefficients (*B)*	Standardized Coefficients (*Beta*)	95% Confidence Interval	*R2*	*P*
ΔRDV-(40–53)				0.1156	0.0408*
Intercept	-0.3394	-	(-0.5635, -0.1153)		0.0033*
ΔTrefoil (vertical)	0.7026	0.0768	(-1.2358, 2.6411)		0.4739
ΔComa (vertical)	-0.0546	-0.0148	(-0.8699, 0.7607)		0.8947
ΔComa (horizontal)	-0.3670	-0.0691	(-1.4464, 0.7123)		0.5016
ΔTrefoil (horizontal)	1.4276	0.1242	(-0.9518, 3.8070)		0.2369
ΔSA	-1.8916	-0.2909	(-3.2328, -0.5505)		0.0061*
ΔHOA	-0.4673	-0.1128	(-1.2456, 0.3110)		0.2365
ΔRDV-T				0.1650	0.0037*
Intercept	-0.4670	-	(-0.7051, -0.2289)		0.0002*
ΔTrefoil (vertical)	0.5575	0.0557	(-1.5027, 2.6176)		0.5927
ΔComa (vertical)	-0.3007	-0.0746	(-1.1672, 0.5659)		0.4930
ΔComa (horizontal)	2.3418	0.4028	(1.1946, 3.4889)		0.0001*
ΔTrefoil (horizontal)	1.0612	0.0844	(-1.4676, 3.590)		0.4072
ΔSA	-0.519	-0.073	(-1.9443, 0.9064)		0.4719
ΔHOA	0.2532	0.0559	(-0.5739, 1.0804)		0.5452
ΔRDV-N				0.1817	0.0015*
Intercept	-0.1733	-	(-0.4164, 0.0699)		0.1606
ΔTrefoil (vertical)	-0.2275	-0.022	(-2.3307, 1.8758)		0.8306
ΔComa (vertical)	0.1714	0.0413	(-0.7132, 1.0561)		0.7016
ΔComa (horizontal)	-2.0513	-0.3421	(-3.2225, -0.8802)		0.0007*
ΔTrefoil (horizontal)	0.8423	0.0650	(-1.7393, 3.4240)		0.5191
ΔSA	-1.1814	-0.1611	(-2.6366, 0.2738)		0.1104
ΔHOA	-0.4315	-0.0923	(-1.2760, 0.4129)		0.3133

Note: Δ, postoperative value minus preoperative value; RDV, refraction difference value; RDV-N, RDV-nasal; RDV-T, RDV-temporal; TRDV, total RDV of 0 to 53°; SA, spherical aberration; HOA, total higher-order aberrations.

* P<0.05.

## Discussion

With the development of science and technology and the continuous innovation of corneal refractive surgery equipment, personalized kerato-refractive surgery and higher surgical precision and postoperative optical quality have gradually become the focus of attention. SMILE procedure uses a femtosecond laser photodissociation blasting to cut directly between the corneal layers and remove the lenticule through a small incision, which avoids the lifting of the corneal flap and maintains the stability of the anterior corneal surface structure and corneal biomechanics [15]. The safety and effectiveness of SMILE surgery have been fully verified and affirmed by clinical practice.

In this study, we determined the changes in RDV after SMILE surgery compared with before surgery. We found that hyperopic defocus values [TRDV, RDV-(20–53), RDV-S, RDV-T, and RDV-N] in the LM group and hyperopic defocus values [TRDV, RDV-(20–53), RDV-S, and RDV-N] in the HM group were significantly lower at 3 months postoperatively than preoperatively. This finding indicates that SMILE significantly reduced retinal peripheral hyperopic defocus values in the 20° to 53°, superior, and nasal ranges in both groups. Because refractive surgery reduces peripheral retinal hyperopic defocus, the optical focus of the peripheral retina shifts from the posterior part of the retina before surgery to the retina or closer to the retina, improving the imaging quality of the peripheral visual field. Therefore, we may be able to assess a patient’s visual function after refractive surgery with a peripheral defocus examination.

Our study also found no significant differences in TRDV, RDV-(0–53), RDV-S, RDV-I, and RDV-N between the two groups before SMILE surgery. However, the RDV-(40–53), RDV-S, and RDV-N were lower in the HM group than in the LM group after operation. This result indicated that the HM group had a greater reduction in retinal peripheral hyperopic defocus than the LM group. The eye’s optics and the retinal shape contribute to the peripheral refraction [16]. Surgical correction of myopia and corneal wound healing processes may lead to changes in peripheral refraction. Kim et al [17] reported that SMILE resulted in significantly more corneal cutting at the center, midperiphery, and periphery and more corneal flattening at the periphery compared to FS-LASIK. Ye et al. [18] reported that after SMILE, epithelial hyperplasia increased with the increase in corrected SE in the central and paracentral zones of the cornea, while the correlation was opposite in the peripheral zone. A more pronounced change in curvature caused by higher myopic correction would result in a more prominent epithelial thickening within the central optical zone. Conversely, at the outer edge of the optical zone, significant epithelial thinning occurs to restore a smooth corneal surface [19]. Therefore, remodeling in the central and paracentral cornea may affect peripheral refractive outcomes of SMILE. Further in-depth studies and investigations are needed to verify these findings.

SMILE focuses on the corneal stroma with a femtosecond laser, shapes and takes out the stroma lenticule according to the type and degree of refractive error, and finally changes the curvature of the central area of the cornea to correct various refractive errors [20]. Similar to SMILE, OK lenses also correct myopia by changing the curvature of the central area of the cornea [21]. Some studies used MRT to examine RDV in myopic patients wearing OK lenses and found that both TRDV and RDV-(15–53) were significantly lower in children in the OK lenses-wearing group [22, 23]. Similar to the range of eccentricity changes in the above-mentioned studies, both TRDV and RDV-(20–53) were significantly lower after SMILE in this study. The formation of myopia is closely related to the retinal defocal state and AL [24, 25]. The OK lenses can control myopia by reducing the retinal peripheral hyperopic defocus and delaying the growth of AL [26]. Therefore, for special adolescent myopic patients with high myopia and high astigmatism, rapid myopic progression, and high retinal peripheral defocus values, but who are not suitable for conventional myopic control methods, whether it is possible to use corneal refractive surgery to reduce the retinal peripheral defocus for myopic control remains unclear. This research gap may become the direction of myopia control research in special adolescent myopic patients.

Wavefront aberrations are phase distortions of light entering the eye that cause defects in image formation, thus reducing the quality of vision [27, 28]. Detection of wavefront aberration provides an objective method to evaluate postoperative optical quality. In this study, we applied iTrace analyzer to measure the corneal higher-order aberrations and total ocular wavefront aberrations before and 3 months after the operation. We found that aberrations [trefoil (vertical), coma, and HOA] in the LM group and aberrations (trefoil, coma, SA, and HOA) in the HM group were significantly higher at 3 months postoperatively than preoperatively, showing that SMILE introduces some surgically induced higher-order aberrations. Similar to our findings, Xia et al. [29] found that the HOA and vertical coma were significantly higher after 7 years of SMILE correction of moderate to high myopia than before operation. Yu et al. [30] observed that HOA, SA, coma, and trefoil (aberrations) increased 1 year after SMILE compared with before operation. Our study also found that trefoil (horizontal) and SA did not change significantly in the LM group but showed an upward trend in the HM group after operation. There were no significant differences in trefoil, coma, and SA before the operation between the LM and HM groups. Postoperatively, coma, trefoil (horizontal), SA, and HOA were higher in the HM group than in the LM group. These results indicate that more higher-order aberrations were introduced in the HM group than in the LM group. This finding is similar to the research results by Jin et al. [31]. Their study found significantly more surgically induced aberrations in the HM group than in LM group after SMILE surgery, and changes in total corneal HOAs, especially vertical coma and SA, were related to the SE. These findings may be related to the changes in corneal morphology caused by the surgery. The surgical incision, postoperative centration and periphery wound healing, and asymmetry of the eye plane such as inclination, decentration, and irregularity, might influence the induction of aberrations [32–34]. The SMILE-induced epithelial remodeling involves both epithelial thickness and epithelial thickness inhomogeneity, is associated with preoperative and treatment parameters, and exerts a significant impact on corneal HOA alterations [35]. Since more central corneal tissue was removed in the HM group than in the LM group, this may increase the deformation of corneal morphology. The above factors may have introduced more surgically induced higher order aberrations in the HM group.

We further investigated the relationship between changes in RDV and those in aberrations after SMILE surgery. In the simple linear regression, some linear relationships were observed between RDV in different regions and changes in aberrations. In the multivariate analysis, we found that ΔRDV-(40–53) was significantly correlated with ΔSA, ΔRDV-T was significantly correlated with Δcoma (horizontal), and ΔRDV-N was significantly correlated with Δcoma (horizontal). As the changes in retinal peripheral defocus were significantly associated with the changes in aberrations, especially SA and coma (horizontal), they can somewhat reflect the visual quality of patients after SMILE. Through preoperative examination, we can design individualized corneal refractive surgery guided by retinal peripheral defocus topography for patients with high retinal peripheral hyperopic defocus values. In addition, RDV examinations using MRT may become a valuable complementary measurement for assessing the optical quality after refractive surgery.

In summary, our findings suggest that the SMILE procedure reduces retinal peripheral hyperopic defocus but introduces some higher-order aberrations, which are more pronounced in people with high myopia refractive errors. However, the results of this study are limited by the small sample size and the short observation time. Therefore, further in-depth studies with a longer follow-up time and a larger sample size are needed to confirm the present findings.

## Supporting information

S1 Table The variation of the RDV in the LM and HM group before and after SMILE. Note: LM, Low and Moderate Myopia; HM, High Myopia; RDV, refraction difference value; RDV-I, RDV -inferior; RDV-N, RDV-nasal; RDV-S, RDV-superior; RDV-T, RDV-temporal; TRDV, total RDV of 0 to 53°. *: P<0.05.
(DOCX)

S2 Table The variation of the wavefront aberrations in the LM and HM group before and after SMILE. Note: LM, Low and Moderate Myopia; HM, High Myopia. SA, spherical aberration; HOA, total higher-order aberrations. *: P<0.05.
(DOCX)

S1 Data (XLSX)
