# Supplementary material for: Changes in relative peripheral refraction and optical quality in Chinese myopic patients after small incision lenticule extraction surgery
Source: PLoS One. 2023 Oct 4;18(10):e0291681. doi: 10.1371/journal.pone.0291681 (PMC10550148; doi:10.1371/journal.pone.0291681)
Supplement: S1 Table — Note: LM, Low and Moderate Myopia; HM, High Myopia; RDV, refraction difference value; RDV-I, RDV -inferior; RDV-N, RDV-nasal; RDV-S, RDV-superior; RDV-T, RDV-temporal; TRDV, total RDV of 0 to 53°. *: P<0.05. (DOCX) [file pone.0291681.s001.docx]

**S1Table**

**The variation of the RDV in the LM and HM group before and after SMILE**

| RDV |  | Preop | Post 3mo | t1/u1 | p1-value |
| --- | --- | --- | --- | --- | --- |
| TRDV | LM group(n=66) | 0.67 ± 0.42 | 0.32 ± 0.46 | 5.8252 | ＜0.0001* |
|  | HM group(n=46) | 0.53 ± 0.45 | 0.16 ± 0.54 | 5.3051 | ＜0.0001* |
|  | t0 | 1.6957 | 1.6802 |  |  |
|  | p0-value | 0.0933 | 0.0965 |  |  |
| RDV-(0–10) | LM group(n=66) | 0.00 ± 0.03 | 0.00 ± 0.03 | -0.4774 | 0.6347 |
|  | HM group(n=46) | -0.01 ± 0.04 | 0.02 ± 0.05 | -3.8585 | 0.0004* |
|  | t0 | 1.2409 | -1.8342 |  |  |
|  | p0-value | 0.2179 | 0.0706 |  |  |
| RDV-(10–20) | LM group(n=66) | -0.03 ± 0.08 | -0.06 ± 0.12 | 1.7918 | 0.0778 |
|  | HM group(n=46) | -0.03 ± 0.10 | -0.03 ± 0.13 | ＜0.0001 | ＞0.9999 |
|  | t0 | 0.3424 | -1.0479 |  |  |
|  | p0-value | 0.7330 | 0.2973 |  |  |
| RDV-(20–30) | LM group(n=66) | 0.18 ± 0.22 | -0.05 ± 0.31 | 5.298 | ＜0.0001* |
|  | HM group(n=46) | 0.21 ± 0.25 | 0.03 ± 0.30 | 4.1121 | 0.0002* |
|  | t0 | -0.6552 | -1.4658 |  |  |
|  | p0-value | 0.5140 | 0.1459 |  |  |
| RDV-(30–40) | LM group(n=66) | 0.51 ± 0.39 | 0.18 ± 0.47 | 4.3052 | 0.0001* |
|  | HM group(n=46) | 0.49 ± 0.46 | 0.21 ± 0.54 | 4.0325 | 0.0002* |
|  | t0 | 0.2129 | -0.2939 |  |  |
|  | p0-value | 0.8319 | 0.7695 |  |  |
| RDV-(40–53) | LM group(n=66) | 0.94 ± 0.57 | 0.56 ± 0.70 | 3.2705 | 0.0017* |
|  | HM group(n=46) | 0.94 ± 0.73 | 0.25 ± 0.88 | 6.1034 | ＜0.0001* |
|  | t0 | -0.0202 | 2.0097 |  |  |
|  | p0-value | 0.9839 | 0.0477* |  |  |
| RDV-S | LM group(n=66) | 0.9 ± 0.57 | 0.19 ± 0.68 | 6.2798 | ＜0.0001* |
|  | HM group(n=46) | 0.68 ± 0.67 | -0.20 ± 1.01 | 7.1987 | ＜0.0001* |
|  | t0 | 1.7909 | 2.2839 |  |  |
|  | p0-value | 0.0768 | 0.0253* |  |  |
| RDV-I | LM group(n=66) | 0.42 ± 0.51 | 0.46 ± 0.69 | -0.4069 | 0.6855 |
|  | HM group(n=46) | 0.32 ± 0.53 | 0.24 ± 0.76 | 0.8128 | 0.4206 |
|  | t0 | 0.9805 | 1.5790 |  |  |
|  | p0-value | 0.3293 | 0.1178 |  |  |
| RDV-T | LM group(n=66) | 1.19 ± 0.58 | 0.80 ± 0.64 | 3.5027 | 0.0008* |
|  | HM group(n=46) | 0.81 ± 0.77 | 0.90 ± 1.06 | -0.6476 | 0.5205 |
|  | t0 | 2.8179 | -0.6239 |  |  |
|  | p0-value | 0.0061* | 0.5348 |  |  |
| RDV-N | LM group(n=66) | 0.74 ± 0.68 | 0.41 ± 0.84 | 2.3873 | 0.0199 |
|  | HM group(n=46) | 0.58 ± 0.52 | -0.18 ± 0.83 | 6.7262 | ＜0.0001* |
|  | t0 | 1.3645 | 3.7309 |  |  |
|  | p0-value | 0.1752 | 0.0003* |  |  |

Note: LM, Low and Moderate Myopia; HM, High Myopia; RDV, refraction difference value; RDV-I, RDV -inferior; RDV-N, RDV-nasal; RDV-S, RDV-superior; RDV-T, RDV-temporal; TRDV, total RDV of 0 to 53°. *：P<0.05.
