# Supplementary material for: Changes in relative peripheral refraction and optical quality in Chinese myopic patients after small incision lenticule extraction surgery
Source: PLoS One. 2023 Oct 4;18(10):e0291681. doi: 10.1371/journal.pone.0291681 (PMC10550148; doi:10.1371/journal.pone.0291681)
Supplement: S2 Table — Note: LM, Low and Moderate Myopia; HM, High Myopia. SA, spherical aberration; HOA, total higher-order aberrations. *: P<0.05. (DOCX) [file pone.0291681.s002.docx]

S2Table

The variation of the wavefront aberrations in the LM and HM group before and after SMILE

| Aberrations |  | Preop | Post 3mo | t1/u1 | p1-value |
| --- | --- | --- | --- | --- | --- |
| Trefoil(vertical) | LM group(n=66) | 0.07 ± 0.06 | 0.09 ± 0.06 | -2.4261 | 0.0180* |
|  | HM group(n=46) | 0.09 ± 0.06 | 0.09 (0.04, 0.19) | 331.5 | 0.0363* |
|  | t0/u0 | -1.9166 | 1316.5 |  |  |
|  | p0-value | 0.0583 | 0.2345 |  |  |
| Coma(vertical) | LM group(n=66) | 0.06 (0.03, 0.1) | 0.20 ± 0.13 | 97.5 | ＜0.0001* |
|  | HM group(n=46) | 0.10 ± 0.07 | 0.35 ± 0.32 | -5.0688 | ＜0.0001* |
|  | t0/u0 | 1260.5 | -2.8941 |  |  |
|  | p0-value | 0.1285 | 0.0054 |  |  |
| Coma(horizontal) | LM group(n=66) | 0.06 ± 0.04 | 0.12 ± 0.08 | -5.8236 | ＜0.0001* |
|  | HM group(n=46) | 0.04 (0.02, 0.08) | 0.28 ± 0.20 | 70 | ＜0.0001* |
|  | t0/u0 | 1671 | -5.0016 |  |  |
|  | p0-value | 0.367 | ＜0.0001* |  |  |
| Trefoil(horizontal) | LM group(n=66) | 0.05 ± 0.04 | 0.06 ± 0.05 | -1.6563 | 0.1025 |
|  | HM group(n=46) | 0.05 ± 0.05 | 0.1 ± 0.08 | -3.8846 | 0.0003* |
|  | t0 | 0.7056 | -2.407 |  |  |
|  | p0-value | 0.4823 | 0.0189* |  |  |
| SA | LM group(n=66) | 0.12 ± 0.05 | 0.12 ± 0.06 | -0.8505 | 0.3982 |
|  | HM group(n=46) | 0.11 ± 0.03 | 0.27 ± 0.15 | -7.1241 | ＜0.0001* |
|  | t0 | 0.5422 | -6.3241 |  |  |
|  | p0-value | 0.5888 | ＜0.0001* |  |  |
| HOA | LM group(n=66) | 0.29 ± 0.11 | 0.36 ± 0.13 | -3.9133 | 0.0002* |
|  | HM group(n=46) | 0.42 ± 0.14 | 0.54 ± 0.24 | -2.7272 | 0.0091* |
|  | t0 | -5.4864 | -4.5083 |  |  |
|  | p0-value | ＜0.0001* | ＜0.0001* |  |  |

Note: LM, Low and Moderate Myopia; HM, High Myopia. SA, spherical aberration; HOA, total higher-order aberrations. *：P<0.05.
